# 2758. Clinical Significance and Antifungal Susceptibility Profile of 103 Clinical *Scedosporium* Species Complex and *Lomentospora prolificans* Isolated from NIH Hospitalized Patients

**DOI:** 10.1093/ofid/ofad500.2369

**Published:** 2023-11-27

**Authors:** Mary Czech, Frida Stock, Chioma Aneke, Mihalis Lionakis, Jennifer Cuellar-Rodriguez, Amir Seyedmousavi

**Affiliations:** National Institute of Allergy and Infectious Diseases, Bethesda, Maryland; NIH, Rockville, Maryland; Microbiology Service, Department of Laboratory Medicine, Clinical Center, National Institutes of Health, Bethesda, Maryland; NIH, Rockville, Maryland; National Institute of Allergy and infectious Diseases, Bethesda, MD; Clinical Center, National Institutes of Health, Bethesda, Maryland

## Abstract

**Background:**

Members of the fungal genera *Scedosporium* species complex and *Lomentospora* have been increasingly recognized as emerging opportunists affecting immunocompromised patients. Reduced susceptibility to systemic antifungals is common, and optimal treatments are incompletely described.

**Methods:**

103 clinical isolates from NIH, Bethesda, MD, were investigated. The identity of each isolate was confirmed at the species level via PCR-sequencing of internal transcribed spacer (ITS) of the ribosomal DNA (rDNA) region and the calmodulin gene. Antifungal susceptibility testing was conducted in accordance with the CLSI M38-A3 guidelines. Patient data were collected retrospectively.

**Results:**

The frequency of *Scedosporium* species complex and *Lomentospora prolificans* isolates are detailed in Figure 1. In vitro susceptibility results of eight antifungals against all isolates are listed in Table 1. The novel antifungal olorofim showed the lowest MICs against all *Scedosporium* and *L. prolificans*, followed by micafungin. Among triazoles, voriconazole (VRC) showed lower MIC values against clinical members of *Scedosporium*. Amphotericin and posaconazole (POS) demonstrated species-specific and inter-species variable activity. Itraconazole, isavuconazole, and terbinafine had high MICs against *Scedosporium* and *L. prolificans*.

Clinical data were available for 90 isolates. 9 patients (28 isolates) had disease or infection (Table 2). All but one case occurred in immunocompromised hosts, and all patients were treated with a regimen that included VRC or POS. Five patients died. Three patients with chronic granulomatous disease were cured following hematopoietic cell transplant. 24 patients (62 isolates) had colonization, of which 58 isolates reflected respiratory colonization in patients with bronchiectasis.

**Figure:**
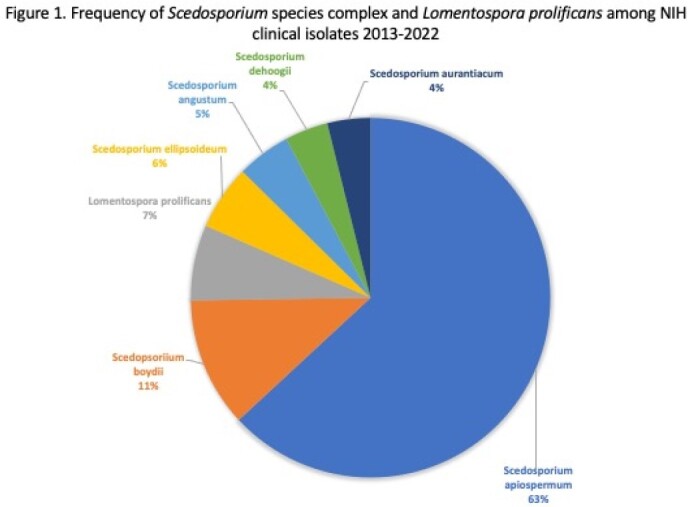

**Figure:**
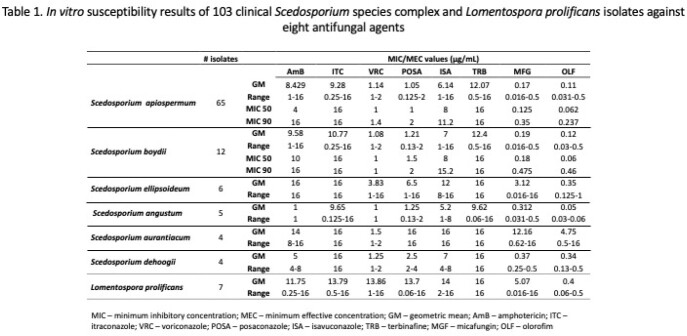

**Figure:**
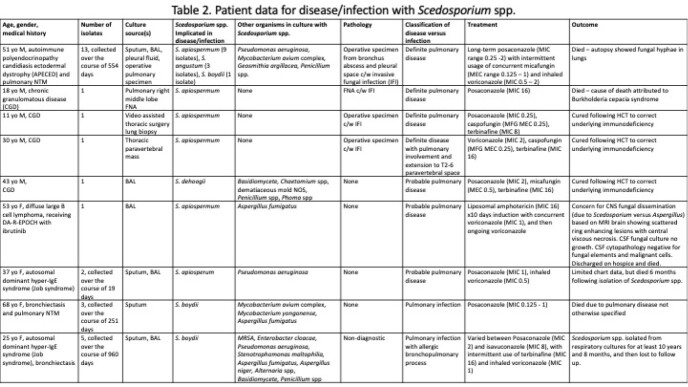

**Conclusion:**

Our data support that species-specific and inter-species differences exist in the distribution of antifungal susceptibility patterns among *Scedosporium* and L. *prolificans*. Our in vitro data suggest that olorofim may be a promising therapy for *Scedosporium* and *L. prolificans*. Our clinical data suggest that host status, in conjunction with effective antifungal therapy, is an important determinant in treatment outcomes.

**Disclosures:**

**All Authors**: No reported disclosures

